# Whey Protein Isolate Film and Laser-Ablated Textured PDMS-Based Single-Electrode Triboelectric Nanogenerator for Pressure-Sensor Application

**DOI:** 10.3390/s22062154

**Published:** 2022-03-10

**Authors:** Minwoo Lee, Jonghwan Shin, Sunkook Kim, Srinivas Gandla

**Affiliations:** Multifunctional Nano Bio Electronics Lab, Department of Advanced Materials and Science Engineering, Sungkyunkwan University, Suwon 16419, Korea; alchemist@skku.edu (M.L.); jhshin0118@skku.edu (J.S.); seonkuk@skku.edu (S.K.)

**Keywords:** whey protein isolate, PDMS, laser ablation, triboelectric nanogenerator

## Abstract

The use of biopolymers for realizing economical and eco-friendly triboelectric nanogenerators (TENGs) widens the application prospects of TENGs. Herein, an animal-sourced whey protein isolate (WPI) film, processed and prepared by a simple aqueous solution preparation and drop-casting technique, is applied to demonstrate its potential use in bio-TENGs. With the addition of formaldehyde in WPI, the films result in a free-standing and flexible film, whereas the pure WPI films are difficult to handle and lack flexibility. A TENG device based on the WPI and the laser-ablated textured polydimethylsiloxane (PDMS) for pressure-sensor application were developed. The output voltage of the TENG comprising WPI increased nearly two-fold compared to the TENG without WPI. A simple single-electrode TENG device configuration was adopted so that it could be easily integrated into a wearable electronic device. Moreover, WPI film exhibited tribo-negative-like material characteristics. This study provides new insights into the development of biocompatible and eco-friendly biopolymers for various electronic devices and sensors.

## 1. Introduction

Biopolymers using eco-friendly, sustainable, clean, and renewable natural materials that are biocompatible and/or biodegradable are crucial for any kind of electronics or materials research field [1,2]. These polymers in electronic devices can substantially eliminate the global problem of electronics waste, leading to a friendly environment. Biopolymers have been successfully implemented in various electronics fields to replace conventional synthetic polymers. Recently, biopolymers have been widely applied in triboelectric nanogenerators (TENGs) [3,4,5,6]. TENGs are devices that convert any form of mechanical energy available in the environment into electrical energy [7]. Their uses are not limited to energy generation as they are also useful for self-powered sensing [8,9,10,11,12]. Examples of this are self-powered sensors based on modified TENG device configurations and materials such as device structure, rough surfaces, and the incorporation of nanomaterials. After the invention of TENGs by Zhong Lin Wang et al. in 2012, many synthetic polymers, including polypropylene, polyvinyl chloride, polystyrene, nylon, Teflon, and polyurethanes, typically derived from petroleum oil have been employed in TENGs. Most recently, to realize a greener environment, polysaccharide-based biopolymers such as cellulose, chitosan, starch, and lignin have been employed as dielectrics for Bio-TENGs [13,14,15,16]. On the other hand, protein-based biopolymers obtained from animals and plants are relatively economical because of their availability as by-products [17]. However, the extraction and purification processes of these biopolymers are complex and time-consuming [6,18,19,20]. Therefore, exploring new biopolymers that are straightforward and economical in terms of both processing and fabrication has become of crucial importance for successful use in TENG devices. Whey protein isolate (WPI), a by-product of cheese production, is commercially available as a powder and is economical, edible, and widely used in the food industry [17]. In addition, WPI films processed in the food industry offer flexibility, transparency, and oxygen barrier properties. Moreover, the commercially available WPI powder products are soluble in an aqueous solution, and they could possibly be used to form films with a simple drop-casting method. Although water-soluble gelatin protein has been reported, a film with freestanding ability has not been shown [21]. Aqueous solution-processed plant-based proteins have shown interesting results to enhance crop yield and quality [22]. Note that pure protein films lack film-forming properties; therefore, they require plasticizers and crosslinking agents to attain flexibility [20]. Thus, whey protein biomaterial for TENGs could certainly enable the development of low-cost devices for various sensing applications. They can even be explored based on their functional material attributes.

Herein, an aqueous solution-processed WPI biopolymer film has been successfully applied in a TENG as a positive triboelectric material. WPI cross-linked with sufficient amounts of formaldehyde resulted in a freestanding, flexible, and transparent film. In many reports, the potential use of biopolymers is demonstrated by combining an opposite triboelectric material in a top-down two-electrode TENG device configuration. Besides, a single-electrode TENG configuration avoids a complex device design. Generally, opposite triboelectric materials with a relatively large difference in the ability to lose or gain charge produce high output performance. Among several triboelectric materials tested with the optimized WPI film, PDMS, a negative triboelectric material, showed better performance. PDMS is a well-known elastomer to exhibit elastic-like properties within the elastic region. Therefore, to have a related application combining WPI and the PDMS pressure sensor application was demonstrated. The WPI film attached to an adhesive aluminum (Al) tape combined with laser-treated textured polydimethylsiloxane (PDMS) elastomer exhibited enhanced triboelectric performances compared to the device without WPI film, which emphasizes the role of WPI film.

## 2. Materials and Methods

### 2.1. Materials

Commercially available WPI with protein content >90% from Hilmar 9410, Hilmar Ingredients, (Hilmar, CA, USA), glycerol from LG Household & Health Care (Seoul, Korea), and formaldehyde solution obtained from Sigma-Aldrich (St. Louis, MO, USA) were purchased and used as received. Glycerol was used as a plasticizer to overcome the brittleness of WPI films.

### 2.2. Materials Characterization

Contact angle measurement: The surface hydrophilicity of the WPI films was measured by contact angle measurement (SmartDrop, Femtobiomedinc, Seongnam, Korea) using a sessile drop method. Deionized water of 5 µm droplets was dropped onto the surface of the WPI films using a precision syringe. The characteristic image of the contact angle was captured within 10 s of the release using an inbuilt software, and its profile was resolved. The contact angle between the solid-to-liquid baseline and the tangent at the drop boundary of the gas-liquid interface was measured at different positions on the WPI surface.

FTIR spectroscopy: FTIR was used to investigate the chemical composition of the WPI film using Nicolet™ iS™ 50 (Thermo Scientific, Waltham, MA, USA). Infrared spectrum analysis was performed over a wavenumber range of 500–4000 cm^−1^.

UV–visible spectroscopy: The transmittance of the WPI films was measured with a UV–visible spectrophotometer (Cary5000, MEMS Sensor Platform Center of Sungkyunkwan University, Suwon, Korea) in the UV and visible range of 200–800 nm. The transmittance value corresponding to a wavelength of 600 nm is considered the transparency of the film.

Tensile measurement: The tensile modulus or Young’s modulus values of the WPI films were measured from the inverse of the slope of the stress versus the strain curves obtained with a Mark 10 test stand using a force gauge with a load cell of 250 N at 50 mm/min. The measurements were performed at room temperature.

### 2.3. Film Preparation

WPI powder (10% *w*/*w*) was dissolved in deionized (DI) water. After the complete dissolution of the powder, glycerol (10% *w*/*w*) and formaldehyde (0%, 20%, 40%, 60%, and 100% *w*/*w*) were added to the aqueous WPI solution. The obtained solution mixtures with different formaldehyde concentrations were constantly stirred for 24 h for hydration and homogenization. For film formation, 4 mL of the final WPI solution was cast on a Teflon surface (35 × 75 mm) and allowed to dry in an ambient atmosphere for at least 48 h. The samples were then heated at 60 °C for 1 h to remove excess water and then peeled off accordingly. A nanosecond IR laser with a wavelength of 1064 nm was used to irradiate the WPI/AgNWs interface for appearance.

### 2.4. TENG Device Preparation

The as-prepared WPI film was peeled off and attached to the Al adhesive tape, which served as a dielectric/bottom electrode for a single-electrode TENG device. To enable a pressure-sensing mechanism, a PDMS elastomer, a promising negative triboelectric material opposite to the WPI, was selected. It is known that an increase in surface roughness, that is, an increase in the surface-to-volume ratio, increases the triboelectric charge upon contact. Therefore, the PDMS surface was exposed to a laser to create a rough morphology. The rough surface has spike-like structures with a size of a few hundred micrometers. After the laser treatment, the PDMS film was dipped in IPA and ultrasonicated for 5 min to remove unwanted debris. The thickness of all the WPI films was ~180 µm. Finally, the laser-treated PDMS surface with a rough surface facing the WPI placed on the WPI film completes the pressure sensor TENG device.

### 2.5. Electrical Characterization

A digital storage oscilloscope (Keysight InfiniiVision DSOX1102G, Santa Rosa, CA, USA) was used to record the electrical voltages of the TENG device. A Mark-10 digital force gauge (model ESM303, Mark 10 Corp., Copiague, NY, USA), capable of tension and compression force up to 250 N, was used to record the applied forces on the TENG device. For example, the device was placed on a force gauge fixed upside down to a supporting stand and a load was manually applied with a metal block (covered with PDMS) on the device. This procedure was repeated for all the electrical measurements of the TENG device.

## 3. Results

**Figure 1:**
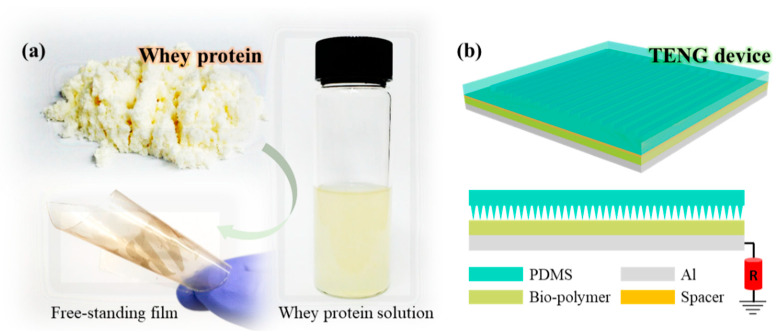
Concept image of the workflow. (**a**) Whey protein isolate powder dissolved in DI water and its freestanding film after preparation. (**b**) Single electrode Bio-TENG pressure device.

**Figure 2:**
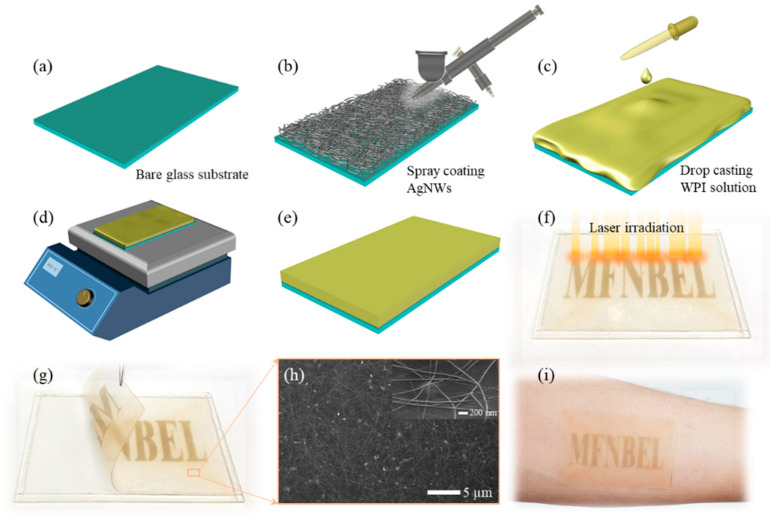
(**a**–**e**) Fabrication of freestanding transparent AgNWs/WPI film. (**f**,**g**) Laser irradiation on AgNWs for appearance followed by peel-off. (**h**) SEM micrograph of the spray-coated AgNWs. (**i**) AgNWs/WPI film transferred onto the human skin.

Figure 1 shows a conceptual image of the WPI film for a TENG-based pressure sensor device. The process of forming a freestanding film of WPI from its commercially available powder form and a subsequently prepared solution is presented in Figure 1a. A TENG device consisting of a WPI film and a laser-processed textured PDMS film for pressure-sensor application is shown in Figure 1b. Details regarding the materials and solution preparation are discussed in Section 2, Materials and Methods. Figure 2a–e schematically illustrate the process flow of preparing the WPI film with spray-coated silver nanowires (AgNWs) at the bottom of the film. Figure 2f shows the highlighted laser beam drawings and a real image of the WPI film after laser irradiation. A real image of the AgNW/WPI film during the peeling off from the Teflon tape (attached to the glass) and the SEM micrograph (inset showing the enlarged image) corresponding to the underlying WPI film are shown in Figure 2g,h. The laser-written AgNWs/WPI film attached to the forearm skin after spraying a liquid bandage (Nexcare) is shown in Figure 2i.

**Figure 3:**
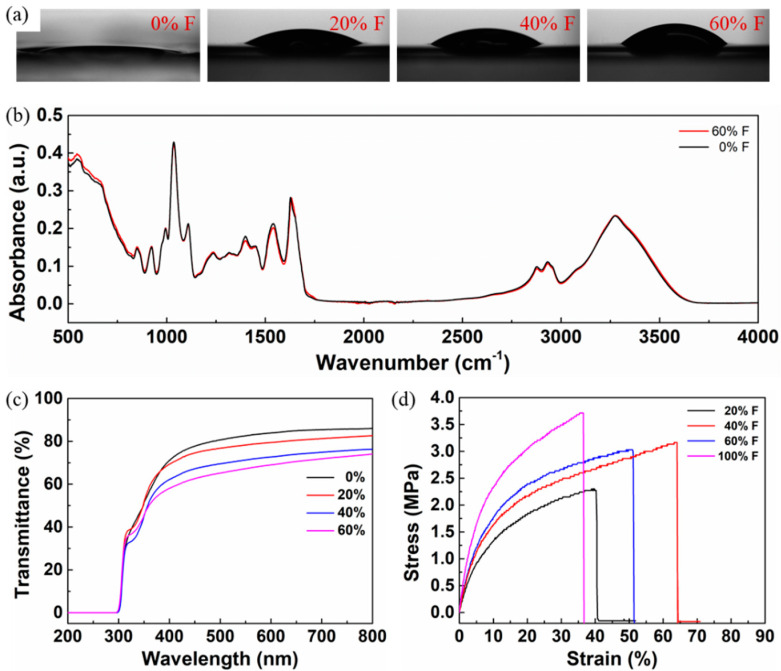
(**a**) Contact angle, (**b**) FTIR spectrum, (**c**) transmittance spectrum, and (**d**) tensile measurements of WPI films with different concentrations of formaldehyde.

The contact angle (CA) images of the WPI films containing formaldehyde of different concentrations, 0%, 20%, 40%, and 60% (*w*/*w*), are shown in Figure 3a. It is explicit from the CA images that with an increase in the concentration of formaldehyde, the contact angle increased up to 42.65° for 60% formaldehyde. The decrease in the surface energy trend with the increase in formaldehyde concentrations may be due to the enhanced crosslinking of buried WPI hydrophobic groups with the formaldehyde [23]. It was also verified that adding acyl group to the casein and WPC increased the surface hydrophobicity [24]. Even more hydrophobicity of the films can be achieved with a heated whey protein solution to induce molecular unfolding that allows protein-protein interactions with the internal hydrophobic groups that are involved in the formation of a network-like matrix [25]. It was deduced that the incorporation of formaldehyde in WPI decreased the surface energy of the film.

The Fourier transform infrared spectroscopy (FTIR) spectrum of the WPI films containing 0% and 60% formaldehyde is presented in Figure 3b. The presence of formaldehyde in the WPI film did not affect the chemical structure or form new bonds. In WPI films, with and without formaldehyde, the spectral intensities did not follow a similar trend, which may be due to the heterogeneity of the protein composition in the films. The spectral range between 800–1150 cm^−1^ is associated with the absorption of glycerol (plasticizer) bands related to C-O and C-C vibrations. The spectral region of 1200–1350 cm^−1^ is related to amide III with N-H in-plane bending and C-N stretching vibrations. The peaks at 1650 and 1590 cm^−1^ are attributed to the peptide bonds (CO-NH) corresponding to the C=O and C-N vibrations of Amide-I and N-H and the C-N vibrations of Amide-II groups. These peaks are closely associated with the concentration of protein and are also responsible for distinguishing the type of proteins present within the samples [26,27,28]. The spectral region from 2800 to 3000 cm^−1^ is assigned to the C-H stretching vibrations of carbonyl groups. The broad peak in the region of 3000–3600 cm^−1^ corresponds to the stretching of -OH groups and N-H stretching. This bandwidth is related to the tendency or degree of cross-linking of proteins driven by hydrogen bonds; therefore, the lower the amount of -OH groups, the higher the degree of crosslinking of proteins with the nearest chains [29]. It was confirmed that all the spectral peaks and regions coincide well with the reported WPI-based films.

A transmittance spectrum of the WPI films with different concentrations of formaldehyde is shown in Figure 3c. Transparency of the WPI films was determined by the transmittance percentage (T%) value at a wavelength of 600 nm. The WPI film of 60% (*w*/*w*) formaldehyde has nearly 70% transmittance. It was observed that the transparency of the WPI films decreased with an increase in the concentration of formaldehyde; however, the reason behind the decrease in transparency is unclear.

The tensile properties of the WPI films containing glycerol (10% *w*/*w*) and different concentrations of formaldehyde (20%, 40%, 60%, and 100% *w*/*w*) are shown in Figure 3d. All the WPI films with formaldehyde content showed a higher tensile modulus and elongation at break than the film without formaldehyde. Note that the film without formaldehyde content was difficult to handle and failed to possess flexible film-like properties. With the increase in the formaldehyde concentration from 20% to 100%, the tensile modulus increased from 23.2 MPa to 48 MPa. It was verified that amino acid in protein forms a covalent bond with the formaldehyde within the proximity of the proteins because of the smaller molecular size of formaldehyde [30]; consequently, there occurs a higher degree of crosslinking in the protein-to-protein network. However, the amino groups were shown to have less access to formaldehyde crosslinking because of the native tertiary structure of proteins; thus, higher concentrations of formaldehyde were needed to crosslink effectively [31]. The elongation at the breaks of all the films did not follow a similar trend, which might be due to the heterogeneity of the protein network in the films. Hence, films prepared from WPI, glycerol, and formaldehyde are flexible and stronger than those prepared from pure WPI.

## 4. Discussion

### TENG Pressure Sensor

**Figure 4:**
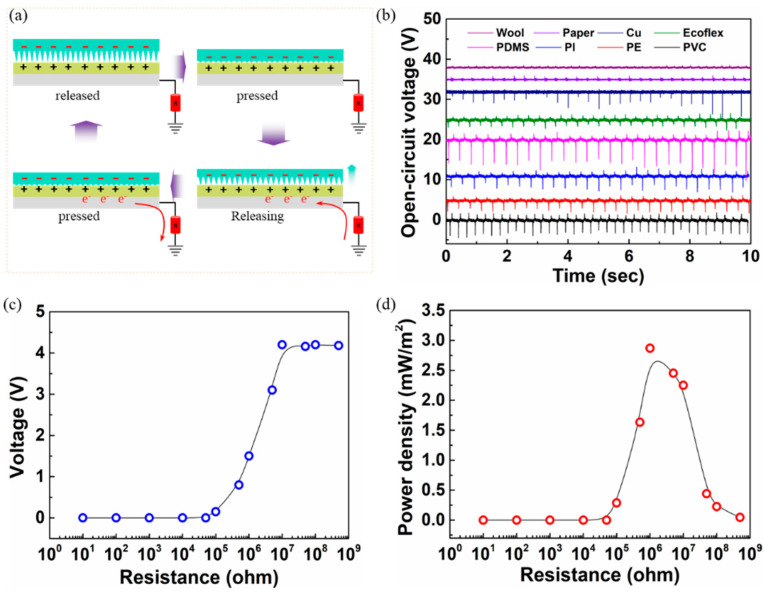
Bio-TENG pressure sensor device structure and characteristics. (**a**) Schematic representation of the TENG pressure sensor under various applied pressures and released conditions. (**b**) The open-circuit voltage output of the bio-TENG device with different positive and negative materials upon contact and release cycling. (**c**,**d**) Output voltage and power density at various load resistances.

The device structure of the single-electrode TENG-based pressure sensor is shown in Figure 4a. The device consists of a WPI film attached to an adhesive Al tape and a surface textured PDMS film. The surface of the PDMS film was textured to increase the effective surface area to enable pressure-sensitive behavior. The surface had a periodic spike-like morphology with features height and base width of 400 µm and 300 µm, respectively, distributed throughout the active area of the surface. The WPI film pressed against the surface-textured PDMS substrate exhibited an open-circuit voltage (*V_oc_*) and short-circuit current (I_SC_) higher than those of the WPI film pressed with the planar PDMS counterparts, wherein the enlarged contact surface area between the two materials tends to induce a greater amount of surface charges on the WPI, as per the following equation:

$$V_{oc}=-\frac{\sigma A}{2C_{0}}$$

where *σ* is the induced surface charge density, *A* is the effective contact area between the two materials, and $C_{0}$ is the capacitance of the TENG device. The basic principle of the Bio-TENG-based pressure sensor can be understood from previous studies [32,33,34,35,36]. Initially, under the no-pressure condition, the two different insulating materials at rest have neutral charges; upon applying pressure, a chemical bond is formed at the interface of the two materials, and the transfer of charge occurs to balance their electrochemical potential.

**Figure 5:**
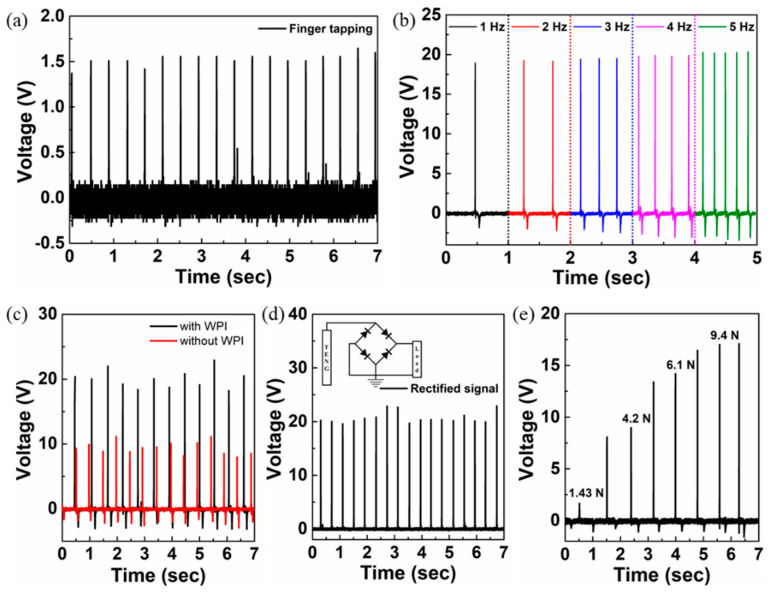
Output voltages of the TENG device under various circumstances: (**a**) finger tapping, (**b**) different frequencies, (**c**) with and without WPI film, (**d**) rectified Wheatstone bridge connection, and (**e**) various loads of the bio-TENG device.

Upon applied pressure, the electrons at the WPI surface are transferred to the PDMS surface because of their work function differences, attaining positive and negative triboelectric charges at the respective surfaces. This process of building up electrostatic charges continues significantly to a certain extent of pressure value and later saturates owing to the structural elastic limit of the pressure-sensitive PDMS film. As the transfer of charges maximizes to an upper limit of applied pressure and upon any amount of pressure release, there are a resultant definite number of electrostatic charges between the two surfaces of materials. This results in a substantially equivalent amount of potential difference generated between the electrodes because the triboelectric charges on both sides of the surfaces are separated by a gap. In return, a potential drop caused by the effective triboelectric charges induces opposite electrical charge carriers in the WPI electrode, during which current flows from the WPI electrode to the ground opposite to the electron flow. In the released condition, induced by the triboelectric charges on the WPI surface, an effective number of electrons reaches the single electrode with no further flow of current between the two electrodes. Again, further pressing the textured PDMS substrate decreases the electric field (reducing the potential difference) between the triboelectric charges, which eventually reduces the induced opposite charge carriers and, therefore, the flow of electrons back to the ground with current flowing into the single electrode. The performance of the single-electrode WPI-TENG device was examined using a series of materials, including polyvinyl chloride (PVC), polyethylene (PE), polyimide (PI), polydimethylsiloxane (PDMS), Ecoflex, copper (Cu), paper, and wool (Figure 4b). Depending on the type of material used for triboelectrification, the tendency of a material to lose electrons or gain electrons classifies it as a positive or negative material, respectively. When two oppositely classified materials undergo contact, the amplitude and polarization of the voltage depend largely on the ability of the materials to lose or attract electrons. The larger the number of electrostatic charges induced between the two opposite materials, the higher the output voltage, *V_oc_*. From the triboelectric series, materials such as PVC, PE, PI, PDMS, and Ecoflex are more likely to gain an electron and, therefore, become more tribo-negative in the increasing order. In contrast, positive materials such as glass, Al, paper, and wool are less likely to gain electrons, showing weak electrostatic induction with the WPI film. It was confirmed that the materials in contact with the WPI film agreed well with the triboelectric series. The output power of the Bio-TENG device was evaluated by varying the load resistance in the circuit (Figure 4c,d). The maximum output voltage and power density of 4 V and 2.8 mW m^−2^, respectively, were obtained at a load resistance of 1 MΩ. The voltage response of the WPI-TENG under finger tapping (without a glove) at a pressure of approximately 2 kPa is shown in Figure 5a. A slight increase in voltage with an increase in the applied frequency under the contact-separation mode is observed, which might be due to the increased pressure and reduced number of neutralized charges with the increased frequency (Figure 5b). The output voltage of the TENG device with and without WPI film is shown in Figure 5c. The increase in voltage in the case of the WPI film may be due to the reduced charge neutralization compared to without the WPI film. Figure 5d shows the rectified (positive) signal using the Wheatstone bridge connection of the diodes connected to the Bio-TENG device. With an increase in load, an increase in voltage is observed, which is due to the increased surface contact area at the interface (Figure 5e).

**Figure 6:**
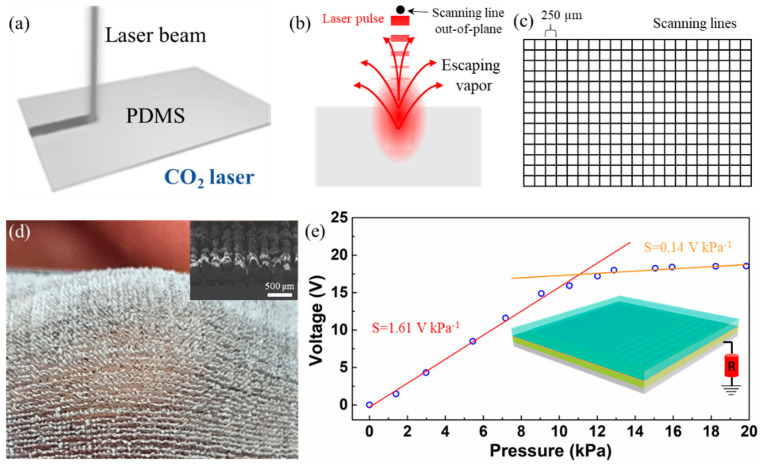
(**a**) Schematic showing the laser beam interacting with the PDMS surface and (**b**) a laser ablation technique to remove the PDMS surface corresponding to one laser scanning line. (**c**) An array of vertical and horizontal scanning lines with 250 µm gaps. (**d**) Real image of the laser-ablated PDMS surface with inset showing the SEM micrograph. (**e**) Pressure sensitivity under mechanical loading of the bio-TENG-based pressure sensor device.

The TENG-based pressure sensor is accomplished by combining a laser-ablated PDMS surface with the WPI film. Unlike photolithography and template-assisted techniques, the laser ablation technique offers additional benefits such as by being rapid, facile, mask independent, low cost, and user friendly [37]. Instead of an IR laser, a CO_2_ laser was subjected to ablate the PDMS surface. The reason is that PDMS material shows high transmittance to IR wavelength of 1064 nm, which would be difficult to process. On the other hand, PDMS shows high absorption to a 10.6 µm wavelength of a CO_2_ laser. The schematic representation of the laser interaction with the PDMS surface is shown in Figure 6a and its corresponding laser ablation showing the escaped vapors of PDMS under temperatures reaching above its melting point during laser beam irradiation is shown in Figure 6b. The PDMS surface is laser irradiated with an array of scanning lines (vertical and horizontal) of 250 µm gaps over an area of 9 cm^2^ (Figure 6c). Real and corresponding SEM micrographs are shown in Figure 6d. It is evidenced that the rough profile of the PDMS surface has a spike-like morphology that enables pressure-like sensitivity. The performance of the TENG-based pressure sensor device is shown in Figure 6e.

The sensor showed a linear behavior up to a pressure of 10 kPa and remained almost constant beyond that value. The increase in voltage is due to the increased surface area of the textured PDMS under pressure. This agrees with the extent of pressure where the textured PDMS undergoes elastic deformation (region I, red color-fitted line) and later saturates (structural elastic limit) with no further compression within the material (region II, orange color-fitted line). The structural elastic limit for a typical viscoelastic material depends on the physical parameters and inherent properties of the material. The physical parameters are related to the morphology of the structure, such as shape and dimensions, and inherent properties are related to the chemical composition, such as the modulus and Poisson’s ratio. Diverse materials such as PDMS, Ecoflex, polyurethane, and even biodegradable materials, in various shapes such as pyramids, domes, cones, pillars, and nature-inspired architectures, have been adopted to widen the pressure sensing range for pressure-sensor applications [38,39,40,41]. The single-electrode pressure sensor device exhibited pressure sensitivities of 1.61 V kPa^−1^ and 0.14 V kPa^−1^ in regions I and II, respectively. In addition to PDMS, biodegradable elastomer materials such as poly(glycerol sebacate) and poly(octamethylene maleate (anhydride) citrate) may also be applied for surface texturing by laser ablation technique. The biomaterial and laser ablation techniques proposed in this study can enable wearable TENG devices with added benefits in terms of biodegradability and conformability.

## 5. Conclusions

In summary, an animal-sourced WPI film derived from milk was successfully applied for potential use in bio-TENG. From a simple aqueous-based solution and drop-casting method, films of WPI and formaldehyde are transparent and possess flexible film-like properties with a tensile modulus of 48 MPa; besides, the films without formaldehyde are difficult to handle. The WPI film exhibited tribo-positive material-like characteristics when subjected to mechanical loads with PDMS, a tribo-negative material. Benefitting from the relative higher triboelectric voltage among the WPI and PDMS, a TENG device comprised of WPI and laser-ablated rough PDMS for pressure-sensor application was successfully demonstrated with a maximum sensitivity of 1.61 V kPa^−1^ in the linear region. The TENG’s performance is nearly twice that of the TENG device without WPI film, which shows the potential use of WPI in bio-TENGs. Moreover, the ablated PDMS film by the laser-ablation technique provides great opportunities to produce films with textured surfaces without any additional use of masks and chemicals for etching. The textured PDMS film shows pressure-sensor-like characteristics owing to the increased surface-to-volume ratio of the PDMS surface. With these findings, the WPI film can be applied to other devices for wearable sensor applications.
